# The importance of recognizing faciobrachial dystonic seizures in rapidly progressive dementias

**DOI:** 10.1590/s1980-5764-2016dn1004016

**Published:** 2016

**Authors:** Mateus Mistieri Simabukuro, Paulo Ribeiro Nóbrega, Milena Pitombeira, Wagner Cid Palmeira Cavalcante, Ronnyson Susano Grativvol, Lécio Figueira Pinto, Luiz Henrique Martins Castro, Ricardo Nitrini

**Affiliations:** 1 Neurology Division, Hospital das Clínicas da Faculdade de Medicina da Universidade de São Paulo, SP, Brazil.; 2 Hospital Geral de Fortaleza, Fortaleza, CE, Brazil.

**Keywords:** anti-LGI1 encephalitis, faciobrachial dystonic seizures, autoimmune encephalitis, rapidly progressive dementias, Creutzfeldt-Jakob disease

## Abstract

**Background:**

Creutzfeldt-Jakob Disease (CJD) is the prototypical cause of rapidly progressive dementia (RPD). Nonetheless, efforts to exclude reversible causes of RPD that mimic prion disease are imperative. The recent expanding characterization of neurological syndromes associated with antibodies directed against neuronal cell surface or sympathic antigens, namely autoimmune encephalitis is shifting paradigms in neurology. Such antigens are well known proteins and receptors involved in synaptic transmission. Their dysfunction results in neuropsychiatric symptoms, psychosis, seizures, movement disorders and RPD. Faciobrachial dystonic seizure (FBDS) is a novel characterized type of seizure, specific for anti-LGI1 encephalitis.

**Objective:**

In order to improve clinical recognition we report the cases of two Brazilian patients who presented with characteristic FDBS (illustrated by videos) and anti-LGI1 encephalitis.

**Methods:**

We have included all patients with FBDS and confirmed anti-LGI1 encephalitis and video records of FDBS in two tertiary Brazilian centers: Department of Neurology of Hospital das Clínicas, Sao Paulo University, Sao Paulo, Brazil and Hospital Geral de Fortaleza, Fortaleza, Brazil between January 1, 2011 and December 31, 2015.

**Results:**

Both patients presented with clinical features of limbic encephalitis associated with FBDS, hyponatremia and normal CSF. None of them presented with tumor and both showed a good response after immunotherapy.

**Conclusion:**

FBDSs may be confounded with myoclonus and occurs simultaneously with rapid cognitive decline. Unawareness of FDBS may induce to misdiagnosing a treatable cause of RPD as CJD.

## Abstract (pt)

**Embasamento:**

A doença de Creutzfeldt-Jakob (DCJ) é o protótipo de demência rapidamente progressiva (DRP). No entanto, é imperativo que sejam excluídas causas reversíveis de DRPs que possam simular doença priônica. A recente caracterização de síndromes neurológicas associadas a anticorpos direcionados contra antígenos de superfície neuronal ou sinapse, assim denominadas de encefalites autoimunes, está mudando paradigmas em neurologia. Esses antígenos estão envolvidos na transmissão sináptica, sendo que as disfunções destes podem resultar em sintomas neuropsiquiátricos, psicose, crises epilépticas, distúrbios do movimento e DRP. A crise distônica faciobraquial (CDFB) é um tipo de crise recentemente caracterizada e específica da encefalite anti-LGI1.

**Objetivo:**

Para promover um melhor reconhecimento da doença relatamos os casos de 2 pacientes brasileiros que apresentaram CDFBs (ilustradas com vídeos) associadas à encefalite anti-LGI1.

**Métodos:**

Foram incluídos todos os pacientes com CDFBs e encefalite anti-LGI1 confirmados em 2 centros brasileiros terciários: Departamento de Neurologia do Hospital das Clínicas da Universidade de São Paulo, São Paulo, Brasil e o Hospital Geral de Fortaleza entre 01 de janeiro de 2011 e 31 de dezembro de 2015.

**Resultados:**

Ambos os casos apresentaram quadro clinico típico de encefalite límbica associada a CDFBs e exame do LCR sem alterações. Nenhum caso associou-se à presença de neoplasia e ambos apresentaram boa resposta à imunoterapia.

**Conclusão:**

A CDFB podem ser confundidas com mioclonias e ocorrer simultaneamente com rápido declínio cognitivo, o seu não reconhecimento pode induzir ao diagnóstico errôneo de uma causa potencialmente tratável de DRP como sendo DCJ.

## INTRODUCTION

Unlike the more common dementing conditions that typically progress over years, rapidly progressive dementias (RPDs) can develop subacutely over weeks or months. They pose a challenge to neurologists because prompt, thorough and accurate diagnosis is mandatory given that treatable or even curable diseases can be the cause of RPDs. Data from dementia centers attributes 6.4% to 27% of RPD to potentially treatable etiologies.^1-3^

Autoimmune encephalitis, i.e. encephalitis associated with antibodies against neuronal surface or synaptic antigens, and Creutzfeldt-Jakob Disease (CJD), may present with similar clinical, radiologic, electrophysiologic, and laboratory findings.^4-8^

Antibody testing and response to immunotherapy are reliable tools to confirm diagnosis of autoimmune encephalitis, however, they are not readily accessible and results can take several weeks.^9^

Thus, clinical clues to appropriately help discriminate these two conditions are of paramount importance. For example: hyponatremia, seizures, cerebral spinal fluid (CSF), pleocytosis brain abnormalities on T2-weighted and fluid-attenuated inversion recovery (FLAIR) magnetic resonance imaging (MRI restricted to medial temporal lobes) are not expected in prion diseases yet are typically found in autoimmune encephalitis.

Faciobrachial dystonic seizures (FDBS) is a distinctive adult-onset, high-frequency, very brief and highly specific antiepileptic resistant seizure and almost pathognomonic for anti-leucine-rich-glioma inactivated 1 (LGI1) encephalitis.^10-12^

Because FDBS manifest as sudden, myoclonic-like jerks, they can be easily mistaken for myoclonus, a clinical finding included in all diagnostic criteria of CJD.^13-15^ Hence, FDBS in the context of rapidly progressive dementia can lead to erroneous diagnosis.

In our experience, it is important for neurologists to be familiarized with this novel entity to avoid pitfalls when evaluating patients with RPD.

## REPORT OF CASES

**Figure 1:**
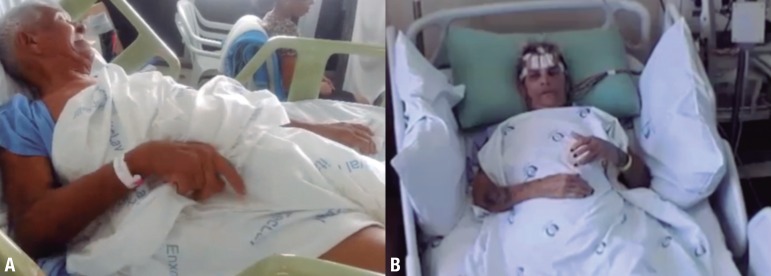
Faciobrachial dystonic seizures (FBDSsW). [A–B] Ictal stills of 2 patients. The ipsilateral face grimacing and arm posturing are visible in both cases. Videos are available in the online supplementary data, with the patients' consent.

**Case 1.** A 72-year-old man presented with progressive cognitive decline and episodes of stereotyped movements of the face and right arm. Four months before the admission, the family noticed progressive apathy, excessive somnolence and difficulty handling money, becoming dependent for basic activities of daily living. He also developed short unilateral jerk movements affecting the right arm and side of face. The episodes lasted a few seconds and initially occurred fifteen times a day. By the time the patient was admitted to the internal medicine ward, he was having these events up to four times per hour (still image Figure 1A, video 1 in supplemental data). At examination, he was disoriented in time and space, scoring 16 on the Mini-Mental State Examination (MMSE) and presented frontal release signs. Routine blood tests revealed mild hyponatremia (128 mg/dL). Brain MRI disclosed T2/FLAIR hyperintensity abnormalities in the right caudate and putamen and left caudate head whereas EEG showed slow activity only. CSF showed mild pleocytosis (10 lymphocytes) with normal glucose and protein content. The patient was referred for neurological assessment with initial diagnosis of Creutzfeldt-Jakob disease.

**Figure 2:**
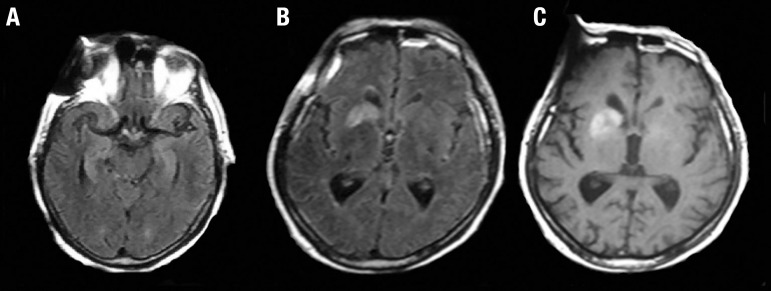
Brain MRI (case 1) showing T2/FLAIR hyperintensities in both mesial temporal lobes [A] and T2/FLAIR and T^1^ hyperintensities in right caudate and putamen [B, C].

A follow-up brain MRI revealed a new hyperintensity on T1-weighted images without contrast enhancement in the right caudate (Figure 2) at the same site of the previous, persisting T2/FLAIR abnormalities. After being evaluated by our center, the presumptive diagnosis of anti-LGI1 encephalitis with FDBS was reached. Both CSF and serum were positive for LGI1 antibodies. Treatment was started with intravenous methylprednisolone (1 g daily for five days) followed by intravenous human immunoglobulin (0.4 g per kilo daily for five days). During the ensuing week, there was only partial improvement of hyponatremia and FBDS, with no improvement in cognition. Given the poor response to first-line treatment, rituximab was started and a significant improvement was seen. At the time of hospital discharge, the patient was alert, scored 19 on the MMSE, hyponatremia was under control and with less than 10 attacks of FDBS per day. The patient was kept on oral prednisone for 6 months with no other chronic suppression.

At long-term follow-up, the patient maintained progressive cognitive improvement, had resumed basic activities of daily living, with no further seizures and stable serum sodium levels. The last MMSE score (at 30 months' follow-up) was 27.

**Case 2.** A 76 year-old woman presented in June of 2015 with subacute onset of forgetfulness, irritability, trouble managing routine daily activities and insomnia. These symptoms worsened and two weeks later she developed focal seizures characterized by brisk and sudden shock-like jerks of her left upper limb. Cognitive complaints progressed to complete compromise of daily tasks in the ensuing weeks. Epileptic seizures with secondary generalization became frequent and she was started on phenytoin.

Two months after symptoms onset she was admitted to Hospital das Clínicas in São Paulo, Brazil. Neurological examination revealed significant cognitive impairment with MMSE score of 19, associated with marked executive dysfunction and frontal release signs. The remaining cognitive domains and somatic neurological exam were normal. Initial diagnostic work-up revealed sodium of 124 mg/dl. A Brain MRI revealed T2/FLAIR hyperintensity in the right hippocampus, right caudate nucleus and putamen. Brain Positron Emission Tomography (PET) showed hypermetabolism in the right hippocampus, prominent hypometabolism in the right caudate and diffusely decreased metabolism in the rest of the cerebral cortex. CSF examination was normal. Further investigation for epileptic seizures was performed with the Video Electroencephalography Monitoring Test (VEEG), which disclosed recurrent, brief and stereotyped dystonic involuntary movements restricted to the left upper limb and ipsilateral face (still image Figure 1 B, video 2 in supplemental data) associated with findings of an ictal electrodecremental pattern. Because of the high suspicion of anti-LGI1 encephalitis and exclusion of alternative causes, empiric treatment with high dose intravenous methylprednisolone and immunoglobulin was performed while awaiting diagnostic tests. Two weeks after start of therapy, a positive result for anti-LGI1 antibodies in the CSF and serum was received.

During follow up, there was resolution of FDBSs episodes, hyponatremia and although her cognitive symptoms improved, she had not returned to baseline level. After 8 months of follow-up, the patient evolved with relapsing symptoms and was retreated with corticosteroids. At last consultation (12 months' follow-up) she scored 22 on the MMSE.

## DISCUSSION

These two cases are illustrative presentations of anti-LGI1 encephalitis with FDBS and rapid cognitive decline. One of these patients (case 1) initially received the diagnosis of CJD, demonstrating the potential for misdiagnosing a treatable condition for another with inexorable fatal progression and no currently available treatment except symptomatic.^16^

The definitive diagnosis of sporadic CJD is reached by obtaining a brain specimen and identifying the protease-resistant PrP^Sc^ deposition with immunohistochemistry or Western blot.^17^ However this approach has many limitations in clinical practice, and CJD diagnosis is based on constellations of symptoms together with paraclinical studies, including biomarkers in cerebral spinal fluid (CSF), EEG and brain MRI (the most important for some authors). The classical clinical feature of CJD is RPD with behavioral abnormalities, ataxia, extrapyramidal features and myoclonus. Therefore, the clinical presentation of these two cases is congruent with prion disease as an important differential diagnosis if the attending physician is unaware of FDBS, a myoclonic-like movement.

Ancillary tests can be deceiving in CJD. For example, despite the specificity for sCJD, periodic sharp wave complexes were recently reported in a case of VGKC encephalitis^7^ and can be present in patients with other etiologies (i.e. Alzheimer disease and vascular dementia).^18^ The utility of CSF biomarkers such as 14-3-3, total tau (t-tau), neuron specific enolase (NSE) is very controversial, as these proteins are not prion specific and the test does not identify prions. Rather than being pathognomonic for CJD, these proteins serve as markers of ongoing neuronal injury.^17^ In recent years, there has been an increasingly number of reports on the lack of specificity of 14-3-3. A Dutch study demonstrated that 50% of patients with autoimmune encephalitis had elevated levels of 14-3-3 in CSF.^19^ Another study demonstrated that 14-3-3 can be found in patients with autoimmune encephalitis.^6^ Even brain MRI, in which abnormal hyperintensity on FLAIR and (diffusion-weighted image) DWI in cortical gyri (cortical ribboning), caudate, putamen or thalamus is considered a very accurate method for diagnosing CJD, has been reported to misdiagnose cases of VGKC encephalitis as CJD.^4,5,20^ Despite the limitation of ancillary testing, a recent test called real-time quacking induced conversion (RT-QuIC) is a very promising premortem diagnostic test.^21^

The importance of being aware of and recognizing FDBS, does not hinge only on distinguishing patients with anti-LGI1 encephalitis and CJD. Although FDBSs are highly specific to anti-LGI1 encephalitis, they precede the onset of cognitive symptoms by weeks in 40-71% of patients.^11,22,23^ For this reason, FBDS are a clue to early diagnosis and immunotherapy with further prevention of cognitive decline and dementia.^24^

The most comprehensive description of FDBS semiology was given in a series of 29 patients.^10^ The FBDS phenomenon is very brief (usually <3 seconds), frequent (median of 50 episodes per day at their peak, ranging from 6-360), always involving the arm and commonly the ipsilateral face (76%) and leg (34%). It can involve either side but is always unilateral within an episode. Patients or bystanders noted ictal loss of consciousness in 66% of patients. Hand involvement can cause many patients to drop objects. Ictal vocalizations occur in 24% and FDBS can be triggered by auditory and emotional stimuli. EEG ictal epileptiform abnormalities were found in a minority of these patients, 7 out of 29, and localization was frontotemporal; frontal or temporal. Response to treatment with antiepileptic drugs only was generally poor and significant side effects were observed in 41% of these patients. Moreover, FDBS response to immunotherapy was excellent. Indeed, in our two cases, satisfactory control of FDBS was achieved only after immunotherapy.

Although described as a distinctive semiology, the epileptic origin of FDBS remains a matter of debate.^25^ Some authors argue that lack of impairment of consciousness and coincident epileptiform activity on ictal scalp EEG together with inconsistent response to different AEDs, points to an alternative, non-epileptic origin.^26^ Interestingly, basal ganglia MRI abnormalities are found in patients with LGI1 encephalitis and FDBS, and are typically contralateral to the episodes, suggesting a subcortical origin.^10,24,27^ This theory is supported by findings of functional neuroimaging (i.e., fluorodeoxyglucose-positron emission tomography or single photon emission computed tomography) studies which disclose altered glucose metabolism in different cerebral regions, including basal ganglia, in most individuals affected.^10,23^

Besides mesial temporal lobe abnormalities (the most known marker of limbic encephalitis), brain MRI of our 2 patients disclosed basal ganglia T1, T2 abnormalities (Figure 2). A Mayo Clinic series of 26 patients with LGI1 encephalitis and FDBS showed that basal ganglia T1 and T2 signal abnormalities were detected in 42% of these patients, including T1 hyperintensity alone (2/11), T2 hyperintensity alone (1/11) or both (8/11).^27^ A separate analysis of all patients with LGI1 encephalitis (with and without FDBS) showed that basal ganglia abnormalities were not present in patients without FDBS and that mesial temporal MRI abnormalities were less common among patients with FBDS than those without. It is noteworthy that there are few causes of basal ganglia T1 hyperintensities (manganese accumulation, CJD, chorea from nonketotic hyperglycemia or lupus, HIV infection, multiple system atrophy, Fahr disease, Wilson's disease, medications such as cyclosporine, Cockayne Syndrome, hypoxic/ischemic injury, intracranial hemorrhage, and multiple MRI scans with gadolinium)^28,29^ compared to T2 hyperintensities (which have a broader list of differential diagnoses) and most are bilateral. Therefore, unilateral basal ganglia T1 hyperintensity is an important marker for anti-LGI1 encephalitis with FDBS. Illustratively, T2/FLAIR abnormalities in the right putamen within the context of a rapidly progressive dementia and myoclonic-like movements led to patient 1 initially receiving a diagnosis of prion disease.

Although a specific marker, FDBS may be present in a minority of patients with anti-LGI1 encephalitis.^11^ Antibodies against LGI1 were discovered in 2010.30,31 Before improved characterization of this target, it was thought that the disease was caused by antibodies directed against voltage-gated potassium channels (VGKC). LGI1 is a unique human epilepsy-related gene that does not encode an ion channel subunit, but is a neuronally secreted protein. It links two epilepsy-related receptors, ADAM22 and ADAM23, in the brain and organizes a transsynaptic protein complex that includes presynaptic potassium channels and postsynaptic alpha-amino-3-hydroxy-5-methyl-4-isoxazolepropionic acid (AMPA) receptor scaffolds.^32^

It is mainly expressed in the hippocampus and temporal cortex. Mutation of the LGI1 protein gene causes autosomal dominant lateral temporal epilepsy.^33^

A Dutch nationwide study documented an annual incidence of anti-LGI1 encephalitis of 0.83/million, which is similar to the incidences of CJD and Lambert Eaton myasthenic syndrome in this country.^11^

Two recent large cohorts of patients with anti-LGI1 encephalitis showed that most patients develop limbic encephalitis, clinically characterized by limbic amnesia, seizures or psychiatric symptoms associated with involvement of medial aspects of temporal lobes on MRI, and CSF pleocytosis or EEG with epileptic or slow activity involving the temporal lobes.^11,34^ (Table 1) Hyponatremia is also an important clue for anti-LGI1 autoimmunity, being present in 60-75% of patients.^11,30,34^ Both of our cases presented with unexplained hyponatremia.

**Table 1:** Clinical features of previously reported series of anti-LGI1 encephalitis cases.

		Irani et al., 2010^31^	Lai et al., 2010^30^	Ariño et. al., 2016^34^	Sonderen et al., 2016^11^
Number of patients		55	57	76	38
Men		37 (67%)	37 (65%)	50 (66%)	25 (66%)
Age at onset, y, median			60 (30-80)	61 (32-80)	64 (31-84)
Limbic Encephalitis		89%	100%	83%	90%
Seizures		89%	82%	67 (88%)	
FBDS before seizures		Not mentioned	Not mentioned	Not mentioned	47%
Hyponatremia		62%	60%	74%	65%
Abnormalities on Brain MRI	Increased T2 signal involving medial temporal lobe(s)	56%	84%	83%	74%
Abnormalities on CSF		Not mentioned	41%	19%	25%
Abnormalities on EEG		Not mentioned	26 (76%)	Not mentioned	50%
Tumor		0%	11%	7%	11%
Treatment	Any	Not mentioned	96%	100%	84%
	First Line (Steroids, IVIg, PLEX)		84%	92%	84%
	Other		12%	56%	Not mentioned
Outcome	Good (mRS 0-2)	91%	78%	71%	67%
Relapses		Not mentioned	6/33 (18%)	27%	35%

FDBS: Faciobrachial dystonic seizures; IVIg: intravenous immunoglobulins; MRI: magnetic resonance imaging; mRS modified Rankin Score; PLEX: plasma exchange; Y: years

Other types of seizure types besides FDBS can also occur in patients with anti-LGI1 encephalitis: focal seizures with mainly dyscognitive, autonomic (goosebumps), motor, gelastic or aura aspects can be present.

In 13% of cases, anti-LGI1 can present with normal brain MRI and CSF without pleocytosis.^34^. Findings of^11,34^ antibodies in CSF or serum confirm a diagnosis of anti-LGI1 encephalitis. Thus far, we recommend testing both serum and CSF because the most recent studies of patients with anti-LGI1 encephalitis have shown conflicting data. The Spanish Group showed that antibodies were detected only in CSF in 8% of patients;^34^ while the Dutch Group showed that CBA in CSF lacks specificity, where only 53% of CSF samples tested positive.^11^ Association with tumor is uncommon in anti-LGI1 encephalitis, and is estimated to be found in 10-11% of patients.^11,30,34^

Although most patients with-LGI1 encephalitis respond to immunotherapy, only 35% are able to return to work or resume all premorbid activities, and 29% remain with moderate-to-severe cognitive problems.^34^ The Dutch cohort showed persistent disturbed spatial recognition memory with normal performance on other memory and neuropsychological tests during follow-up of patients with good recovery.^11^ Case 2 evolved with relapse, which is estimated to occur in 27-35% of patients.^11,34^ Despite a potentially reversible disease with initial improvement after immunotherapy, the outcome of anti-LGI1 encephalitis was far from optimal.

Therefore, it is important to recognize FDBS clinically, since antibody tests are not widely available, and prompt treatment with immunotherapy may shorten the time to recovery and prevent cognitive impairment.

## References

[1] Chitravas N, Jung RS, Kofskey DM, Blevins JE, Gambetti P, Leigh RJ (2011). Treatable neurological disorders misdiagnosed as
Creutzfeldt-Jakob disease. Ann Neurol.

[2] Geschwind MD (2009). Clinical trials for prion disease: difficult challenges, but hope
for the future. Lancet Neurol.

[3] Papageorgiou SG, Kontaxis T, Bonakis A, Karahalios G, Kalfakis N, Vassilopoulos D (2009). Rapidly progressive dementia: causes found in a Greek tertiary
referral center in Athens. Alzheimer Dis Assoc Disord.

[4] Geschwind MD, Tan KM, Lennon VA, Barajas RF Jr, Haman A, Klein CJ (2008). Voltage-gated potassium channel autoimmunity mimicking
creutzfeldt-jakob disease. Arch Neurol.

[5] Yoo J, Hirsch LJ (2014). LImbic encephalitis associated with anti-voltage-gated potassium
channel complex antibodies mimicking creutzfeldt-jakob
disease. JAMA Neurol.

[6] Grau-Rivera O, Sánchez-Valle R, Saiz A, Molinuevo JL, Bernabé R, Munteis E (2014). Determination of neuronal antibodies in suspected and definite
Creutzfeldt-Jakob disease. JAMA Neurol.

[7] Savard M, Irani SR, Guillemette A, Gosselin-Lefebvre S, Geschwind M, Jansen GH (2016). Creutzfeldt-Jakob Disease-Like Periodic Sharp Wave Complexes in
Voltage-Gated Potassium Channel-Complex Antibodies Encephalitis: A Case
Report. J Clin Neurophysiol Off Publ Am Electroencephalogr Soc.

[8] Cavallieri F, Mandrioli J, Tondelli M, Vitetta F, Stipa C, Vallone S (201429). Pearls & Oy-sters: Rapidly progressive dementia: Prions or
immunomediated?. Neurology.

[9] Graus F, Titulaer MJ, Balu R, Benseler S, Bien CG, Cellucci T (2016). A clinical approach to diagnosis of autoimmune
encephalitis. Lancet Neurol.

[10] Irani SR, Michell AW, Lang B, Pettingill P, Waters P, Johnson MR (2011). Faciobrachial dystonic seizures precede Lgi1 antibody limbic
encephalitis. Ann Neurol.

[11] van Sonderen A, Thijs RD, Coenders EC, Jiskoot LC, Sanchez E, Bruijn de MAAM (2016). Anti-LGI1 encephalitis Clinical syndrome and long-term
follow-up. Neurology.

[12] Schmerler DA, Roller S, Espay AJ (2016). Teaching Video NeuroImages: Faciobrachial dystonic seizures
Pathognomonic phenomenology. Neurology.

[13] World Health Organization (1988). Global Surveillance, Diagnosis and Therapy of Human Transmissible
Spongiform Encephalopathies: Report of a WHO Consultation.

[14] Geschwind MD, Josephs KA, Parisi JE, Keegan BM (2007). A 54-year-old man with slowness of movement and
confusion. Neurology.

[15] Zerr I, Kallenberg K, Summers DM, Romero C, Taratuto A, Heinemann U (2009). Updated clinical diagnostic criteria for sporadic
Creutzfeldt-Jakob disease. Brain.

[16] Takada L, Geschwind M (2013). Prion Diseases. Semin Neurol.

[17] Geschwind MD (2015). Prion Diseases. Contin Minneap Minn..

[18] Wieser HG, Schindler K, Zumsteg D (2006). EEG in Creutzfeldt-Jakob disease. Clin Neurophysiol Off J Int Fed Clin Neurophysiol.

[19] Maat P, de Beukelaar JW, Jansen C, Schuur M, van Duijn CM, van Coevorden MH (2015). Pathologically confirmed autoimmune encephalitis in suspected
Creutzfeldt-Jakob disease. Neurol Neuroimmunol Neuroinflammation.

[20] Fermo OP, Izbudak I, Sutter R, Venkatesan A, Kaplan PW, Probasco JC (2014). Autoimmune encephalitis mimicking Creutzfeldt-Jakob
disease. Neurol Clin Pract.

[21] McGuire LI, Poleggi A, Poggiolini I, Suardi S, Grznarova K, Shi S (2016). Cerebrospinal fluid real-time quaking-induced conversion is a
robust and reliable test for sporadic creutzfeldt-jakob disease: An
international study. Ann Neurol.

[22] Shin Y-W, Lee S-T, Shin J-W, Moon J, Lim J-A, Byun J-I (2013). VGKC-complex/LGI1-antibody encephalitis: Clinical manifestations
and response to immunotherapy. J Neuroimmunol.

[23] Irani SR, Gelfand JM, Bettcher BM, Singhal NS, Geschwind MD (2014). Effect of Rituximab in Patients With Leucine-Rich,
Glioma-Inactivated 1 Antibody-Associated Encephalopathy. JAMA Neurol.

[24] Irani SR, Stagg CJ, Schott JM, Rosenthal CR, Schneider SA, Pettingill P (2013). Faciobrachial dystonic seizures: the influence of immunotherapy
on seizure control and prevention of cognitive impairment in a broadening
phenotype. Brain.

[25] Striano P (2011). Faciobrachial dystonic attacks: Seizures or movement
disorder?. Ann Neurol.

[26] Striano P, Belcastro V, Striano S, Irani SR, Schott JM, Vincent A (2011). Tonic seizures: A diagnostic clue of anti-LGI1
encephalitis?. Neurology.

[27] Flanagan EP, Kotsenas AL, Britton JW, McKeon A, Watson RE, Klein CJ (2015). Basal ganglia T1 hyperintensity in LGI1-autoantibody
faciobrachial dystonic seizures. Neurol Neuroimmunol Neuroinflammation.

[28] Ginat DT, Meyers SP (2012). Intracranial Lesions with High Signal Intensity on T1-weighted MR
Images: Differential Diagnosis. RadioGraphics.

[29] Kanda T, Ishii K, Kawaguchi H, Kitajima K, Takenaka D (2013). High Signal Intensity in the Dentate Nucleus and Globus Pallidus
on Unenhanced T1-weighted MR Images: Relationship with Increasing Cumulative
Dose of a Gadolinium-based Contrast Material. Radiology.

[30] Lai M, Huijbers MG, Lancaster E, Graus F, Bataller L, Balice-Gordon R (2010). Investigation of LGI1 as the antigen in limbic encephalitis
previously attributed to potassium channels: a case series. Lancet Neurol.

[31] Irani SR, Alexander S, Waters P, Kleopa KA, Pettingill P, Zuliani L (2010). Antibodies to Kv1 potassium channel-complex proteins
leucine-rich, glioma inactivated 1 protein and contactin-associated
protein-2 in limbic encephalitis, Morvan's syndrome and acquired
neuromyotonia. Brain.

[32] Fukata Y, Lovero KL, Iwanaga T, Watanabe A, Yokoi N, Tabuchi K (2010). Disruption of LGI1-linked synaptic complex causes abnormal
synaptic transmission and epilepsy. Proc Natl Acad Sci.

[33] Morante-Redolat JM, Gorostidi-Pagola A, Piquer-Sirerol S, Sáenz A, Poza JJ, Galán J (2002). Mutations in the LGI1/Epitempin gene on 10q24 cause autosomal
dominant lateral temporal epilepsy. Hum Mol Genet.

[34] Ariño H, Armangué T, Petit-Pedrol M, Sabater L, Martinez-Hernandez E, Hara M (2016). Anti-LGI1-associated cognitive impairment Presentation and
long-term outcome. Neurology.

